# Plasmid pPNptGreen Expression of Green Fluorescent Protein in *Pseudomonas chlororaphis* Strain S1Bt23 Abrogates Biocontrol Activity Against *Pythium ultimum*

**DOI:** 10.1111/1758-2229.70083

**Published:** 2025-04-16

**Authors:** Mercy Akuma, Sylvia Ighem Chi, Renlin Xu, Indira Thapa, Barbara Blackwell, James Tabi Tambong

**Affiliations:** ^1^ Ottawa Research and Development Centre Agriculture and Agri‐Food Canada Ottawa Ontario Canada; ^2^ University of Ottawa Ottawa Ontario Canada; ^3^ Canadian Blood Service Ottawa Ontario Canada; ^4^ Department of Plant Science University of Manitoba Winnipeg Manitoba Canada

**Keywords:** biocontrol, confocal microscopy, green fluorescent protein, phenazines, *Pseudomonas chlororaphis*, pyrrolnitrin, *Pythium ultimum*

## Abstract

*Pseudomonas chlororaphis* is a highly effective plant root coloniser and biocontrol agent. To monitor the colonisation of tomato and canola roots, *P. chlororaphis* S1Bt23 was transformed with the pPNptGreen plasmid encoding for green fluorescent protein (S1Bt23‐GFP). Seedling roots inoculated with S1Bt23‐GFP were examined after 2 and 5 h using confocal laser fluorescence microscopy. Roots exposed to S1Bt23‐GFP showed pronounced biofilm formation around the root surface, and fluorescing cells were localised in the epidermis and metaxylem after 2 and 5 h of inoculation, respectively. The canola roots also showed upward active translocation of the S1Bt23‐GFP cells in xylem vessels in real time. S1Bt23‐GFP was also evaluated for antagonistic activity against *Pythium ultimum* . While S1Bt23 WT exhibited 65.70%–71.4% inhibition of radial growth of *Py. ultimum*, the S1Bt23‐GFP strain did not demonstrate any antagonistic effects. Thin layer chromatography and liquid chromatography mass spectrometry analyses of culture extracts of S1Bt23‐GFP did not detect phenazines or pyrrolnitrin, antifungal metabolites identified in S1Bt23 wild type. Expressions of phenazine and pyrrolnitrin genes showed no differences in S1Bt23‐GFP and wild type. This suggests that the abrogation of these metabolites occurred post‐transcriptionally, probably due to a high cellular molecular load in GFP production. This could negatively impact the ecological fitness of S1Bt23‐GFP.

## Graphical abstract

Transformation of *Pseudomonas chlororaphis* S1Bt23 with plasmid pPNptGreen negates its antagonistic activity against *Pythium ultimum* due to the lack of the production of key secondary metabolites, for example phenazines.

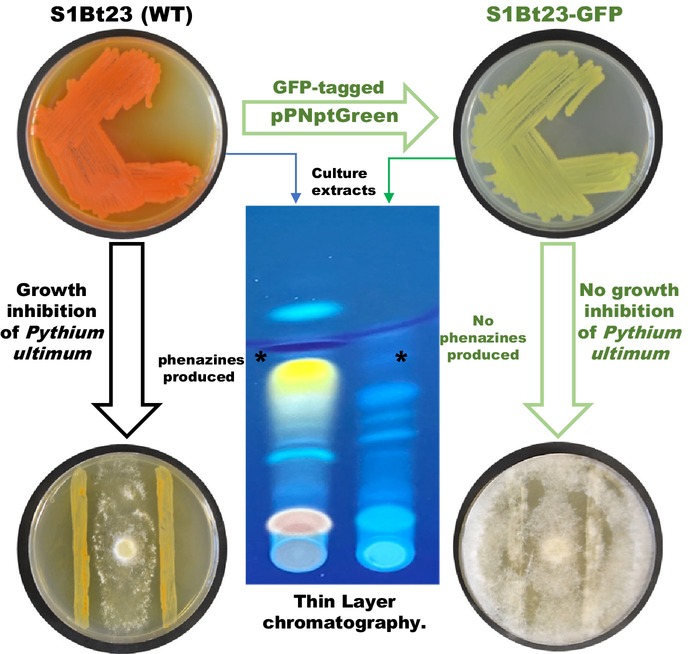

## 1 Introduction

Bacterial biological control agents are becoming strategic alternatives to chemical pesticides for use in the management of major plant diseases. Amongst biocontrol agents, *Pseudomonas* have been reported to have antagonistic activity against a broad range of plant pathogens (Höfte 2021). They are efficient root colonisers and can control soilborne pathogens, making them great candidates for biocontrol (Lugtenberg et al. 2001). In 2005, we isolated a potent strain of *Pseudomonas chlororaphis* , S1Bt23, from woodland soil in Quebec, Canada. In in vitro assays, strain S1Bt23 exhibited potent antagonistic activity against *Fusarium graminearum*, *Pythium ultimum* , *Pythium arrhenomanes*, *Rhizoctonia solani*, *Alternaria alternaria* and *Sclerotinia sclerotium* (unpublished). Whole‐genome sequencing and analysis showed that strain S1Bt23 possesses the complete biosynthetic clusters required for the synthesis of two key antifungal secondary metabolites, phenazines and pyrrolnitrin. The phenazine production is regulated by a conserved set of core biosynthetic genes *phz*ABCDEFG, which catalyses the conversion of chorismic acid to phenazine‐1‐carboxylic acid (PCA) (Biessy and Filion 2018; Diederich et al. 2017). The biosynthesis of pyrrolnitrin is guided by a cluster of four genes, *prn*ABCD, which utilises tryptophan as a precursor for pyrrolnitrin synthesis (Kirner et al. 1998). In addition, thin layer chromatography (TLC) and high performance liquid chromatography (HPLC) assays confirmed the production of these secondary metabolites in growth culture media. This suggests that strain S1Bt23 has potential as a biocontrol agent for application in agricultural ecosystems as a plant disease management strategy. Prior to field applications, it is important to monitor its effectiveness in colonising plant roots.

The use of *gfp* transformants has provided a better method of studying the colonisation and accumulation of bacteria in plants. The traditional method of re‐isolation of soil and root inoculated bacterial cells has significant pitfalls, for example unreliability due to the culturing of non‐target bacteria, laboriousness and time consumption (Liu et al. 2019; Raacke et al. 2006). Green fluorescent protein (GFP) is a versatile tool that has significantly advanced our understanding of how plants interact with microbes. Its non‐invasive nature, coupled with its ability to provide real‐time, dynamic information, makes it an indispensable asset in both basic research and applied fields such as agriculture, biotechnology and environmental science (Harper et al. 1999). The power of *gfp* has allowed plant scientists to visualise specific processes such as gene expression, protein localisation and organelle dynamics in real‐time (Harper et al. 1999; Weill et al. 2019). GFP‐tagged bacteria have been instrumental in studying microbial communities and interactions. By tagging different species or strains with GFP, researchers can track their spatial distribution, population dynamics and interactions within complex microbial ecosystems. GFP‐expressing bacteria can be used as biosensors for environmental monitoring to detect specific pollutants or toxins (Kostrzynska et al. 2002; Rathnayake et al. 2021; Valenzuela‐Garcia et al. 2023) as well as in monitoring the colonisation of plant tissues to better understand the pathogenicity process (Hupp et al. 2019; Lozoya‐Perez et al. 2018; Parente et al. 2008; Wang et al. 2020; Yang et al. 2019). Hupp et al. (2019) used GFP‐transformed *Pseudomonas syringae* to follow its infection in *Arabidopsis thaliana* and reported that the gfp‐transformed *P. syringae* strains induced chlorotic symptoms similar to the non‐marked wild type. Verma et al. (2022) used gfp‐tagged *Escherichia coli* to study colonisation patterns and mutualistic symbiosis in tomato and Bermuda grass seedlings, and the results indicated that the bacterium significantly enhanced root development in seedlings. Also, the biocontrol activities of the endophytic bacteria, *Bacillus amyloliquefaciens* and *Bacillus pumilus*, against the *P. syringae* pv. *tomato* strain NS4 tagged with GFP‐expressing genes have been reported (Filho et al. 2013). Even though gfp tagging of bacteria has provided concrete data on the accumulation and colonisation in plants, little or no data exist regarding potential interference with the production and effectiveness of antifungal secondary metabolites.

The objectives of this study were to (1) tag/mark strain *P. chlororaphis* S1Bt23 with *gfp* using the pPNptGreen plasmid; (2) determine, in vitro, colonisation of canola ( *Brassica napus* ) and tomato ( *Solanum lycopersicum* ) seedling roots and (3) evaluate the antifungal activity of the S1Bt23 *gfp*‐tagged strains. Gfp‐transformed S1Bt23 strains were successfully obtained via triparental mating using pPNptGreen. GFP‐transformed S1Bt23 showed effective colonisation of both the tomato and canola seedling roots. However, the phenotypic properties and metabolic activity of S1Bt23‐GFP cells were significantly different from those of the wild type. Surprisingly, key antifungal metabolites, phenazines and pyrrolnitrin, were not detected in GFP‐transformed S1Bt23 based on Liquid chromatography‐mass spectrometry (LC–MS) and thin‐layer chromatography (TLC) analyses. In addition, S1Bt23‐GFP lost the antagonistic activity against the oomycete *Pythium ultimum* (*=Globisporangium ultimum*), the causal agent of several diseases of major agricultural crops including corn, soybean, potato and wheat. Our findings, therefore, indicate that genetic engineering of biocontrol agents, for example the introduction of *gfp*, may greatly impact the ecological fitness of the strains in the soil environment.

## 2 Results

### 2.1 Development of a GFP‐Expressing *Pseudomonas chlororaphis* S1Bt23 Strain for Visualisation and Colonisation Studies

The in vitro antagonistic activity of S1Bt23 suggested its potential as a biocontrol agent in the field. Therefore, we sought to determine its efficiency in plant root colonisation. In order to visualise S1Bt23 colonisation, we incorporated the plasmid with the gene encoding the green fluorescent protein (*gfp*) into S1Bt23 via triparental mating using helper: *E. coli* strain MC1601 carrying plasmid pRK2013 that promotes mobilisation of the pPNptGreen (GFP reporter) plasmid from donor strain *E. coli* DH5α into recipient strain S1Bt23. pPNptGreen‐positive S1Bt23 transformants were selected by Kanamycin resistance as well as green fluorescence under ultraviolet (UV) light (Figure S1). Observation of S1Bt23‐GFP under UV light confirmed the presence of green fluorescence, an observation not found in cells of the wild type (Figure S1). Colony PCR amplification using specific primers for a 633 bp GFP gene fragment from GFP‐positive strains confirmed the presence of the GFP reporter plasmid in S1Bt23 and the transformants denoted S1Bt23‐GFP (Figure S2). No amplicon was generated in the wild‐type strain (Figure S2). Phenotypically, exogenous expression of GFP led to the loss of the characteristic orange pigment produced by wild‐type S1Bt23. This characteristic orange pigmentation has been associated partly with phenazine‐1‐carboxylic acid. This implied that the incorporation of the pPNptGreen plasmid in S1Bt23 might have induced significant changes in its metabolic activity or profiles.

### 2.2 S1Bt23‐GFP Displays Efficient Tomato and Canola Seedling Root Colonisation

**FIGURE 1:**
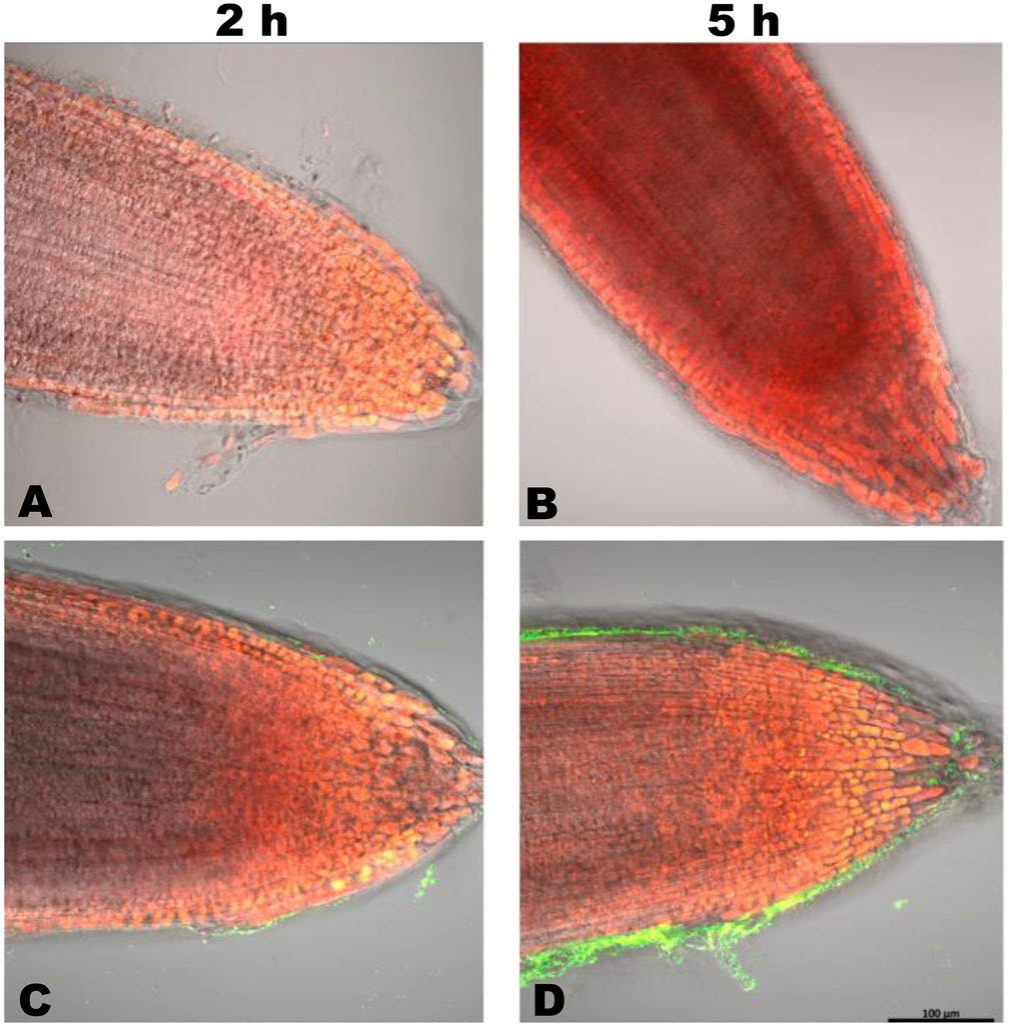
Colonisation of 4‐day old *tomato* seedling root tips by GFP‐transformed *Pseudomonas chlororaphis* S1Bt23 observed and imaged with an LSM 800 (Carl Zeiss Imaging) for untreated (A & B) and S1Bt23‐GFP treated (C & B). Note significant increase after 5 h (D) relative to 2 h (C) exposure time.

**FIGURE 2:**
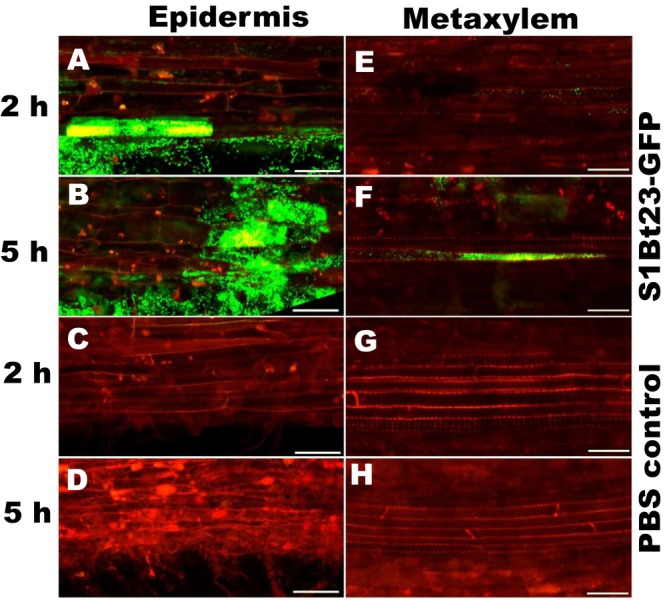
Visualisation of S1Bt23‐GFP cells in the epidermis (left) and metaxylem (right) in 4‐day old tomato seedling roots after 2 and 5 h of exposure time observed and imaged with an LSM 800 (Carl Zeiss Imaging) confocal microscope. Scale bar is 100 μm.

**FIGURE 3:**
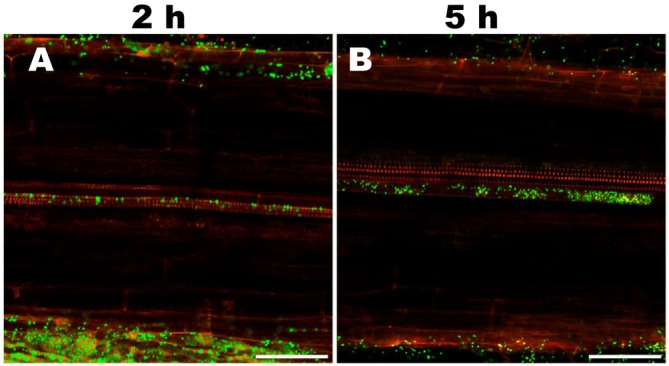
Colonisation of 4‐day old canola seedling roots by *Pseudomonas chlororaphis* S1Bt23‐GFP observed and imaged with an LSM 800 (Carl Zeiss Imaging) using a Fluar 20X objective. GFP‐S1Bt23 could be visualised in the metaxylem vessels after 2 h (A), significantly increased after 5 h (B) of exposure. Scale bar is 100 μm.

To determine plant colonisation by S1Bt23, we inoculated the roots of 4‐day‐old canola and tomato seedlings with the S1Bt23‐GFP strain and monitored bacterial localisation after 2 and 5 h using confocal fluorescence microscopy. Four‐day‐old tomato roots exposed to S1Bt23‐GFP showed pronounced biofilm formation around the root surface and tips on samples incubated for 5 h (Figure 1D) compared to 2 h (Figure 1C). This suggests active attraction of the bacterial S1Bt23‐GFP cells to the rhizosphere. The root surface and tips of control treatments remained intact but with no fluorescence (Figure 1A,B). The S1Bt23‐GFP cells were localised in the epidermis (Figure 2, right column) and metaxylem (Figure 2, left column) after 2 and 5 h of inoculation, respectively. As expected, roots exposed to S1Bt23‐GFP for 2 h (Figure 2A) had significantly fewer fluorescing cells in the epidermis compared to those incubated for 5 h (Figure 2B). The untreated roots did not show any fluorescing cells in either the epidermis (Figure 2C,D) or metaxylem (Figure 2G,H) for both exposure times. Also, no fluorescing cells were detected in the metaxylem after 2 h of exposure (Figure 2E) but were localised after 5 h of inoculation (Figure 2F). Furthermore, 4‐day‐old canola roots exposed to S1Bt23‐GFP showed significant bacterial colonisation in the epidermis and metaxylem, 2 h (Figure 3A) and 5 h (Figure 3B) after incubation. The assay with canola seedlings also depicted the S1Bt23‐GFP cells in the xylem vessels actively being translocated upwardly (Video S1). The rapid and efficient colonisation of tomato and canola roots in vivo by GFP‐transformed S1Bt23 demonstrates its potentially high affinity to colonise these root systems and provide bioprotection of these vital tissues against invading pathogens.

### 2.3 pPNptGreen Tagging of S1Bt23 (S1Bt23‐GFP) Abrogates Biocontrol Against *Py. ultimum*

**FIGURE 4:**
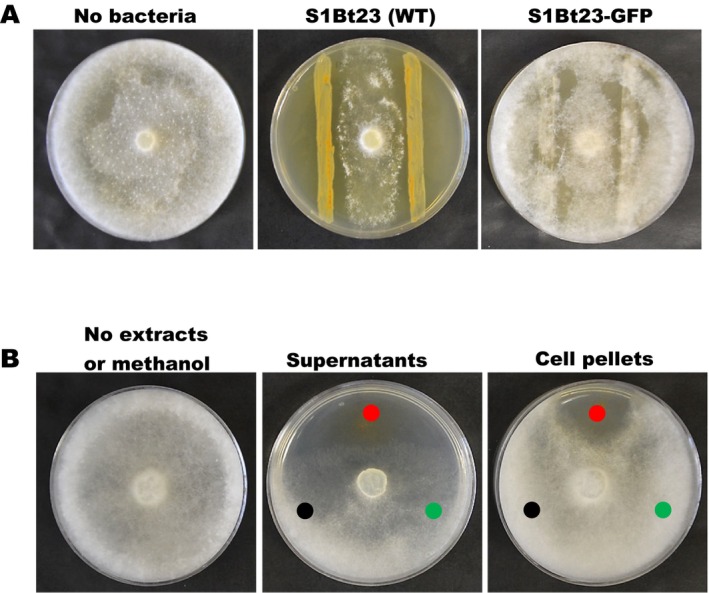
(A) Dual culture assays of *Pythium ultimum* and *Pseudomonas chlororaphis* strain S1Bt23 wild type (WT) or green fluorescent protein‐transformed S1Bt23 (S1Bt23‐GFP). Note the loss of antagonism on glucose‐casamino acid‐yeast agar medium inoculated with S1Bt23‐GFP, 5 days after incubation; and (B) inhibiting effects of metabolic extracts from wild type culture supernatant and cell pellets of S1Bt23 (WT; 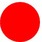), S1Bt23‐GFP (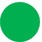) and methanol as solvent control (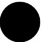). The Petri plate is a negative control with no extracts or methanol.

Given the phenotypic pigment colour changes observed between S1Bt23 wild type (WT) and GFP transformants, we sought to determine if GFP incorporation into S1Bt23 affected its biocontrol activity. Results of dual culture assays involving S1Bt23‐GFP and *Pythium ultimum* on glucose‐casamino acid‐yeast (GCY) medium are shown in Figure 4A. While S1Bt23 WT exhibited 65.70%–71.4% inhibition of radial growth of *Py. ultimum*, the S1Bt23‐GFP strain demonstrated no antagonistic effects (Figure 4A). This suggests that the transformation of S1Bt23 with the pPNptGreen plasmid negatively impacted its antagonistic activity against *Py. ultimum* (Figure 4A).

The culture extracts were analysed to determine whether the loss of biocontrol activity by S1Bt23‐GFP could be due to changes in intracellular and extracellular antifungal metabolites (phenazines and pyrrolnitrin) known to be produced by S1Bt23 wild type. The crude extracts from supernatants and pelleted cells of overnight cultures were tested for phenazines and pyrrolnitrin, respectively. *Pythium ultimum* was incubated on GCY media inoculated with extracts from wild type S1Bt23 and GFP transformants. Intracellular and extracellular extracts from wild type S1Bt23 displayed potent antagonistic activity (Figure 4B; red disc) against *Pythium ultimum* , whereas extracts from S1Bt23‐GFP (Figure 4B; green disc) did not (Figure 4B). Also, the negative or methanol control (black disc) did not exhibit any growth inhibition (Figure 4). This demonstrated that GFP transformation of S1Bt23 with the pPNptGreen plasmid had a negative impact on the production of antifungal metabolites in the GFP‐transformants.

### 2.4 Comparative Analysis of Antifungal Metabolites in *Pseudomonas chlororaphis* S1Bt23 and Its GFP‐Tagged Variant Using TLC and LC–MS

**FIGURE 5:**
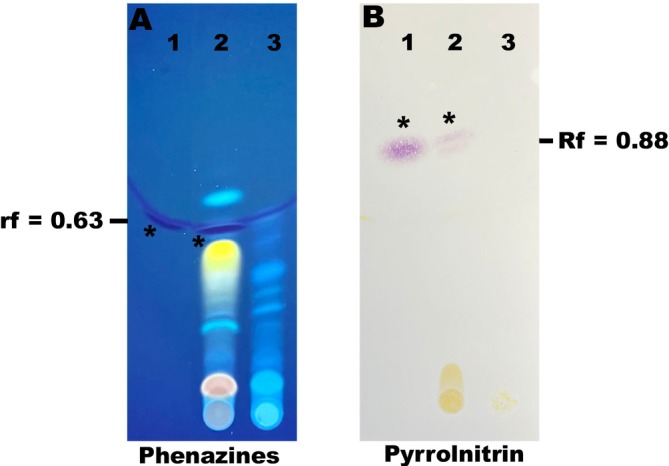
Thin‐layer chromatography (TLC) analysis of extracts from the supernatants (A) and cell pellets (B) wild type (WT) S1Bt23 and S1Bt23‐GFP for the presence of phenazine and pyrrolnitrin, respectively. Synthetic phenazine‐1‐carboxylic acid (PCA) and pyrrolnitrin standards were included. Phenazine visualisation was imaged under UV light and pyrrolnitrin under white light using Erhlich's reagent spray. Note the lack of band depicting either phenazine or pyrrolnitrin in S1Bt23‐GFP: 1, synthetic phenazine or pyrrolnitrin; 2, S1Bt23 (WT); and 3, S1Bt23‐GFP.

Using thin‐layer chromatography (TLC) analysis of extracellular extracts detected the presence of phenazine‐1‐carboxylic acid (Figure 5A; lane 2) and pyrrolnitrin (Figure 5B; lane 2) in S1Bt23 wild type. The retention factors (Rf) of 0.63 and 0.88, respectively, were recorded and are similar to those of the pure synthetic phenazine and pyrrolnitrin (Figure 5). None of these key antifungal compounds were detected in the extracts derived from S1Bt23‐GFP (Figure 5A,B; lane 3).

**FIGURE 6:**
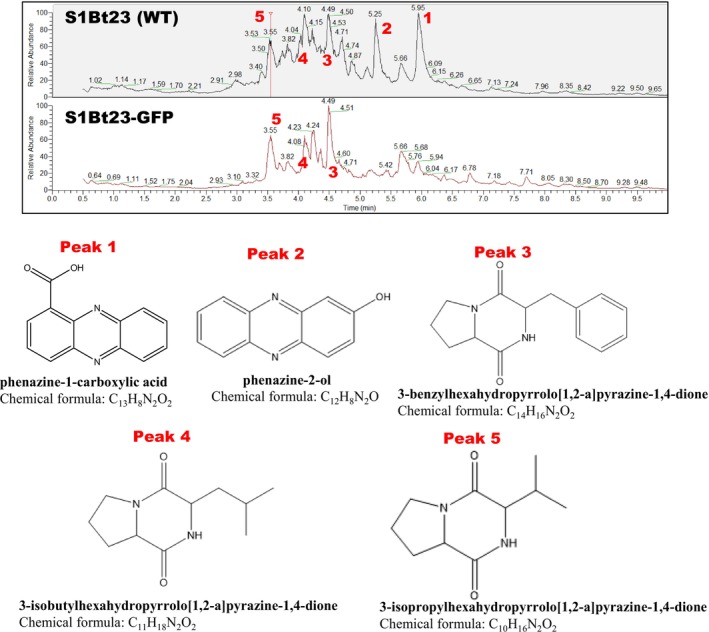
Liquid‐chromatography mass spectrometry (LC–MS) analysis and chemical identification of five metabolic peaks in supernatant extracts of S1Bt23 wild type (WT) and S1Bt23‐GFP reveal the absence of phenazine‐1‐carboxylic acid (peak 1) or phenazine‐2‐ol (peak 2) in the latter strain. Peaks 3–5 are present in supernatant extracts from the wildtype and GFP‐transformants.

Liquid‐chromatography mass spectrometry (LC–MS) analysis confirmed the presence and absence of phenazines in the intracellular and extracellular extracts derived from S1Bt23 wild type and S1Bt23‐GFP, respectively (Figure 6). Five prominent molecular peaks were identified by LC–MS in extracts from S1Bt23 wild type (Figure 6A) but only three similar peaks were found in S1Bt23‐GFP (Figure 6A). We predicted the molecular formulas of the 5 peaks: 1, phenazine‐1‐carboxylic acid, 2, 2‐hydroxyphenazine (= phenazine‐2‐ol), 3, 3‐benzylhexahydropyrrolo[1,2‐a]pyrazine‐1,4‐dione, 4, 3‐isobutylhexahydropyrrolo[1,2‐a]pyrazine‐1,4‐dione, and 5, 3‐isopropylhexahydropyrrolo[1,2‐a]pyrazine‐1,4‐dione (Fig, 6B). Peaks 1 and 2 were identified as phenazine derivatives and were present in S1Bt23 wild type but were absent in S1Bt23‐GFP. Peaks 3, 4 and 5 (Figure 6) were present in extracts of both strains S1Bt23 wild type and S1Bt23‐GFP (Figure 6). The absence of phenazines and pyrrolnitrin in the extract of S1Bt23‐GFP may explain the loss of antagonistic activity against *Py*. *ultimum*.

### 2.5 Gene Expression Analysis Suggests Post‐Transcriptional Disruption of Phenazine and Pyrrolnitrin Biosynthesis in S1Bt23‐GFP Strain

**FIGURE 7:**
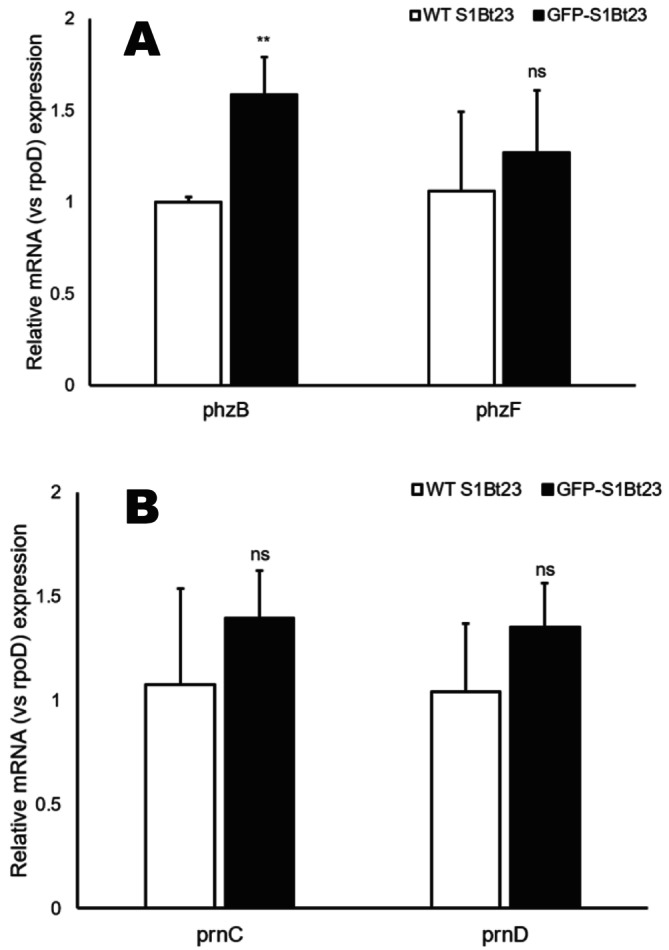
qRT‐PCR analysis of mRNA expressions of *phz*B and *phz*F genes of the phenazine biosynthetic cluster (A) or *prn*C and *prn*D of the pyrrolnitrin cluster (B) in S1Bt23 wild type (WT) and S1Bt23‐GFP. Each gene expression was normalised to the housekeeping gene *rpo*D. The fold change expression of each gene was calculated using the delta–delta Ct method. Statistical significance was calculated using unpaired *t*‐test. **p < 0.01, ns denotes not significant. Error bars represent standard deviation from the mean calculated from three replicates.

The mechanism behind the lack of synthesis and detection of antifungal metabolites is still to be elucidated. We tested the hypothesis that the introduction of the pPNptGreen plasmid into S1Bt23 might have impacted the transcription of genes involved in the biosynthesis of phenazines and/or pyrrolnitrin. Quantitative reverse‐transcriptase PCR analysis of the expression of *phz*F (phenazines) (Figure 7A) and *prn*C and *prn*D (pyrrolnitrin) (Figure 7B) genes showed no statistically significant differences. This suggests that the introduced GFP plasmid did not disrupt the transcription of the key gene clusters involved in the biosynthesis of these antifungal metabolites, an indication that the disruption could be at the post‐transcriptional level (during translation). However, *phz*B, a phenazine gene highly similar to *phz*A, showed a statistically greater expression in S1Bt23‐GFP compared to S1Bt23 wild type.

## 3 Discussion

The increased interest in alternative and sustainable solutions to chemical pesticides in plant disease control has led to more research funding in the discovery of effective biocontrol agents. *Pseudomonas* spp. are well studied in disease management in plants and have been described to control diseases caused by fungal phytopathogens (Mehmood et al. 2023; Saranraj et al. 2023). We isolated a potent *P. chlororaphis* strain S1Bt23 and demonstrated its antagonistic effects against *Pythium ultimum* , the oomycete causal agent of several diseases of major agricultural crops (Stephens and Powell 1982; Del Castillo Munera and Hausbeck 2016). Effective colonisation of the roots by strain S1Bt23 is vital for plant interaction and bioprotection against soilborne diseases (Chin et al. 2001; Haas and Defago 2005). Therefore, we used GFP technology to study the colonisation pattern of the strain S1Bt23 in vitro. GFP is a well‐established tool for understanding gene expression, protein targeting, localisation, and host‐pathogen interactions in intact cells (Allison and Sattenstall 2007; Conn 1999; Kanno et al. 2023). The original green fluorescent protein of *Aequorea victoria* (jellyfish) was discovered in the early 1960s (Shimomura et al. 1962). The cloning of the GFP gene by Prasher et al. (1992) paved the way for use in tracking gene expression in bacteria (Chalfie et al. 1994) and has been genetically engineered to produce a large number of other colour variants (Kremers et al. 2011; Matz et al. 1999; Shaner et al. 2008).

In our study, we transformed strain S1Bt23 with the pPNptGreen plasmid to create a GFP‐expressing strain, S1Bt23‐GFP, allowing us to monitor its colonisation *in planta*. S1Bt23‐GFP effectively colonised the roots of tomato and canola inoculated in vitro. Confocal microscopic analysis revealed significant colonisation of the root tissues of both plants after 2 and 5 h of inoculation. This revealed that strain S1Bt23 is a good coloniser of tomato and canola roots. Effective protection of plants against pathogens and growth promotion by bacterial strains is highly dependent on their colonisation abilities of the target plant root systems (Liu et al. 2016; Zboralski and Filion 2020; Zeriouh et al. 2014). This complex process is known to involve cellular and molecular mechanisms, such as biofilm formation, evasion of plant immunity and biosynthesis of secondary metabolites (Zboralski and Filion 2020). Based on the results of our study, strain S1Bt23 is perhaps genetically equipped for successful colonisation of tomato and canola rhizospheres. The biofilm formation, bacterial aggregates embedded in a hydrated extracellular polymeric substance (Flemming and Wingender 2010), is evident in the results presented especially after 5 h of exposure of tomato and canola roots to strain S1Bt23‐GFP. The development of biofilm depends on chemotaxis, a process essential for plant root colonisation (Hamer et al. 2010; Muriel et al. 2015) suggesting that strain S1Bt23 possesses the core apparatuses/appendages for the perception of key molecular signals. A scan of the whole genome of strain S1Bt23 reveals evidence of an elaborate flagellar protein system, for example *fli*O, *fli*M, *fli*N, *fli*L, *fli*Q, *fli*A, *fle*N, etc., confirming its likely motility. In addition, the whole genome sequence of strain S1Bt23 has all the core chemotaxis genes required for the perception of its chemical environment and trigger movement towards favourable conditions, the root surfaces. The core genes identified include *che*A, *che*B, *che*R, *che*W, *che*Z and *che*V, and are distributed into two distinct operons with the exception of *che*V. *che*V is made up of a *che*W domain that is fused to the domain and a phosphorylatable receiver, and it has been implicated in the modulation of the adaptive response to chemical stimuli (Alexander et al. 2010; Ortega and Zhulin 2016). Strain S1Bt23, thus, has the required cellular arsenal for effective colonisation of plant root surfaces and therhizosphere.

Plant beneficial *Pseudomonas* produce active secondary metabolites to compete with other microbes in the rhizosphere during the colonisation process. Since S1Bt23 biosynthesises phenazine‐1‐carboxylic acid (PCA), an antimicrobial substance, we checked the S1Bt23‐GFP strain for potential antagonism of *Py. ultimum*. The dual culture assay, surprisingly, revealed the inability of S1Bt23‐GFP to antagonise *Py. ultimum*. Since PCA produced by the wild type S1Bt23 has been implicated in the antagonism of this oomycete, the extracts of S1Bt23‐GFP were analysed by TLC, but this active substance was not detected, suggesting that the transformation of S1Bt23 wild type with the pPNptGreen plasmid had abrogated phenazine production. Pyrrolnitrin produced by the wild type was also not detected in extracts of S1Bt23‐GFP. This is the first documented report of the loss of key antifungal secondary metabolites (phenazines and pyrrolnitrin) in a GFP‐transformed strain. Our data, thus, suggest that phenazine and pyrrolnitrin biosynthesis are not essential translational processes in S1Bt23 transformed with the pPNptGreen plasmid. Also, LC–MS analysis identified 3 peaks in extracts of both S1Bt23 wild type and S1Bt23‐GFP with their molecular formulas corresponding to 3‐benzyl, 3‐isobutyl or 3‐isopropyl derivatives of hexahydropyrrolo[1,2‐a]pyrazine‐1,4‐dione. This could suggest that the biosynthesis of these molecules is essential or prioritised even in potentially high metabolic load conditions as a result of GFP expression by plasmid pPNptGreen in S1Bt23. These molecules are reported to be active against biofilm formation by other bacterial competitors such as *Pseudomonas aeruginosa* and *Staphylococcus aureus* (Kiran et al. 2018; Singh et al. 2019).

It is unclear why the loss of biosynthesis of these molecules is observed in S1Bt23‐GFP. Several studies employed GFP to monitor and study root colonisation in sugarcane and rice (Rouws et al. 2010), cotton (Dong et al. 2020; Wang et al. 2020), wheat, maize and cucumber (Hao and Chen 2017) and soybean and Chinese cabbage (Ku et al. 2018) by tagged *Gluconacetobacter diazotrophicus* , *Bacillus subtilis* , *Bacillus axarquiensis* , *Paenibacillus polymyxa* and *Bacillus cereus* , respectively. However, no detrimental effects of the GFP were reported. Also, Hupp et al. (2019) studied the spread and infection of pPNptGreen‐tagged virulent and wild‐type strains of *Pseudomonas syringae* DC3000 in intact *Arabidopsis thaliana* leaves. The study found no difference in the accumulation or “chlorotic halos” symptoms induced by the pPNptGreen‐tagged fluorescing and wild‐type *P. syringae* strains. This could suggest that pathogenicity‐related gene expressions and processes are unsurprisingly essential to *P. syringae* DC3000 or, simply, this pathogenic strain may possess or use other mechanisms to circumvent the considerable metabolic load exerted by GFP production imposed by the pPNptGreen plasmid.

The gene expression analysis of *phz*B and *phz*F, genes involved in phenazine biosynthesis, and *prn*C and *prn*D (involved in pyrrolnitrin biosynthesis) revealed intact transcription, suggesting that the disruption of PCA and pyrrolnitrin synthesis may occur at the post‐transcriptional level. The results of *phz*B and *phz*F (phenazines) and *prn*C and *prn*D (pyrrolnitrin) gene expression studies indicated a robust transcription of these genes and perhaps potential inefficient post‐transcriptional processes. This could be an indication of overexpression of the GFP in the pPNptGreen plasmid due to possibly a very strong promoter, as previously suggested (Bienick et al. 2014), leading to off‐target effects (Allison and Sattenstall 2007; Ganini et al. 2017). For example, overexpression of GFP led to toxicity and interference with the regeneration of transgenic plants (Chiu et al. 1996; Haseloff and Amos 1995). The strong promoter on the pPNptGreen plasmid might have driven a highly significant level of GFP expression, resulting in a considerable cellular metabolic load. This hypothesis was validated by using plasmid pVSP61, a plasmid with the same backbone as pPNptGreen but lacking the *gfp*‐expressing gene, to transform strain S1Bt23. Figure S3 shows identical phenotypes between the S1Bt23 wild type and S1Bt23 tagged with plasmid pVSP61 (S1Bt23‐pVSP61) which differed from that of S1Bt23‐GFP. The antagonistic activity against *Py. ultimum* (Figure S4) and the production of phenazines and pyrrolnitrin, key antifungal compounds, were maintained in S1Bt23‐pVSP61 (Figure S5). Under this metabolic stress condition, the cells of S1Bt23‐GFP possibly had to prioritise their translational processes and resources. It is known that the expression of heterologous protein will typically exert a fitness cost to the host organism, leading to slow growth (Hellmuth et al. 1994; Studier and Moffatt 1986). Scott et al. (2010) proposed the ribosome allocation model in which increased expression of unnecessary heterologous proteins will deprive the cells of resources to synthesise ribosomes.

In conclusion, we transformed strain S1Bt23, a potent antagonist of *Pythium ultimum* , with the pPNptGreen plasmid to visualise its colonisation of tomato and canola roots using confocal microscopy. S1Bt23‐GFP efficiently colonised the root surfaces of both plants within 2 and 5 h after incubation. Fluorescing cells of S1Bt23‐GFP were detected in the epidermis and metaxylem of both plant roots and visualised abundantly after 5 h of incubation. This rapid external and internal colonisation of root tissues is key to enhancing the positive interaction of strain S1Bt23 and plant hosts. However, the incorporation of the plasmid pPNptGreen in S1Bt23 disrupted the production of phenazines and pyrrolnitrin, key antifungal secondary metabolites involved in the antagonistic activity against *Pythium ultimum* . This limits the use of the pPNptGreen plasmid labelling as a tool to study the activity of S1Bt23 under field conditions. The loss of antagonistic activity by S1Bt23‐GFP could also hinder its potential application as an environmental monitoring strategy. Our data suggest that this alteration is more likely to have occurred at the translational level as no significant differences were observed at the transcriptional level. This is the first documented report of the abrogation of phenazine and pyrrolnitrin production in a *P. chlororaphis* strain transformed with a GFP‐expressing plasmid pPNptGreen. Future work will focus on the evaluation and selection of other fluorescence‐expressing systems with lower molecular load on transformed cells of S1Bt23 as well as having minimum adverse effects on the biosynthesis of phenazines and pyrrolnitrin.

## 4 Experimental Procedures

### 4.1 Bacterial Strains, *Pythium* and Growth Conditions

*Pseudomonas chlororaphis* strain S1Bt23 was isolated by (Tchagang et al. 2018) from woodland soil collected from Aylmer, Quebec, Canada. *Escherichia coli* strain DH5a (donor) carrying the plasmid pPNptGreen (green‐flourescent protein (GFP) reporter) was kindly provided by Dr. Bealtie (University of Iowa, USA). The helper strain, *E. coli* strain MC1601 carrying plasmid pRK2013, was provided by Dr. Kirsty Agnoli (University of Zurich, Switzerland). *Pythium ultimum* isolate LevI 805 was provided by Dr. A. Levesque (retired; Ottawa Research and Development Center, Ottawa, Ontario, Canada).

All bacterial strains were grown in Luria‐Bertani (LB) (Sigma, Canada) medium, unless otherwise indicated, at 30°C for *Pseudomonas* strains and 37°C for *Escherichia coli* . Where required, antibiotic concentrations used were 50 μg/mL Kanamycin and 100 μg/mL Ampicillin.

### 4.2 GFP‐Tagging of Strain S1Bt23 With Plasmid pPNptGreen via Triparental Mating

Triparental mating was done as previously reported (Heinze et al. 2018) with some modifications. The OD595 of the cell densities of *E. coli* DH5a (donor), *E. coli* MC1601 (helper) and strain S1Bt23 (recipient) was adjusted to 1.0 and then mixed in a ratio of 1:1:5, respectively, for the triparental conjugation. An aliquot (100 μL) of the mixture was then spotted on an LB agar plate and incubated at 28°C overnight. The cells were collected, and 100 μL of 10‐fold serial dilutions of the culture were plated onto new LB + Ampicillin (50 μg/mL) + Kanamycin (50 μg/mL) plates and incubated overnight at 28°C. Single transconjugant colonies showing GFP expression under UV light were selected for PCR identification with gfp primers. The specific primer set used to confirm the *gfp* gene in selected transformants was 5′‐GGGCACAAATTTTCTGTCAGTGGA‐3′ (forward) and 5′‐CATCCATGCCATGTGTAATCCCAG‐3′ (reverse). GFP‐transformed S1Bt23 colonies were preserved at −80°C until used.

Also, to test the hypothesis that gfp production is loading the bacterial cells, plasmid pVSP61, which has the same backbone as pPNptGreen but lacks the *gfp*‐producing gene, was electroporated into cells of S1Bt23 to obtain pVPS61‐tagged cells (S1Bt23‐pVPS61) as previously described (Hupp et al. 2019). Strain S1Bt23‐pVPS61 was evaluated, in parallel, with S1Bt23‐GFP and S1Bt23 wild type for phenotype, antagonism against *Py. ultimum*, and production of phenazines and pyrrolnitrin.

### 4.3 Bacteria Colonisation and Microscopy Imaging

We evaluated the colonisation of the GFP‐tagged S1Bt23 strain on tomato and canola seedling roots, 4 and 6 days old, respectively. The roots were immersed in the S1Bt23‐GFP bacterial suspension (10^8^ cells/mL) in sterile phosphate saline buffer (PBS) and incubated at room temperature for 2 and 5 h. The negative control treatments consisted of sterile PBS with no bacteria. A direct mounting of the root was done onto the glass slide with Fluoromount G as the mounting medium overlayed with a cover glass. Slides were imaged using the Zeiss LSM800 Laser Scanning Confocal Microscope (Germany) with either the Fluar 20×/0.75 objective or the alpha Plan‐Apochromat 63×/1.46 DIC Oil objective for high‐magnification images or time‐lapse videos. An excitation of 488 nm was used, and bacteria GFP fluorescence was imaged between 490 and 600 nm (pseudo colour green) and the background autofluorescence was imaged between 600 and 700 nm (pseudo colour red). A DIC (Differential Interference Contrast or Nomarski Microscopy) image was taken as well.

### 4.4 In Vitro Dual Culture Assays

Dual culture assays were used to test the antagonistic activity of wild type and S1Bt23‐GFP against oomycetes *Py. ultimum* as previously described (Tchagang et al. 2018). Briefly, a plug of oomycete culture, about 5 mm in diameter, was transferred from a 2‐day‐old agar plate to the center of a potato‐dextrose agar plate or glucose‐casamino acid‐yeast (GCY; glucose 15 g/L, casamino acids 1.5 g/L, yeast extract 1.0 g/L, KH_2_PO_4_ 1.5 g/L, MgSO_4_.7H_2_O 1.0 g/L, agar 15 g/L) agar. Bacteria were streaked at equidistance on both sides of the oomycete plug, incubated at 30°C, and monitored daily. A positive control plate with no bacteria was also included. Inhibition rate (%) was calculated using the formula: ([*G*_f_−*G*_f+b_]/*G*_f_) × 100, where *G*_f_ is the radial growth distance of the oomycete alone, and *G*_f+b_ is the oomycete radial growth distance towards the streaked bacteria.

### 4.5 Metabolite Extraction and Detection Using TLC and LC–MS

Protocol for extraction of antifungal metabolites was adopted from Mehnaz et al. (2009). Wild type and GFP‐S1Bt23 strains were cultured in 200 mL of LB broth at 225 rpm for 90 h at 30°C. Bacterial supernatants were separated from the pellet by centrifugation. Chloroform (30 mL) was added to 200 mL of bacterial supernatant, mixed thoroughly, and allowed to separate into layers. The upper (aqueous) phase was obtained and acidified to pH 3 with HCl. Chloroform was then used to extract organic compounds from the aqueous phase, and the organic phase was allowed to evaporate to obtain solid residues. The crude extracts were dissolved in methanol and stored briefly at 4°C. The bacterial pellet was washed once with phosphate‐buffered saline (PBS) and the metabolites were extracted using methanol: water: chloroform (2:2:1, v/v/v). Intact bacteria were removed by centrifugation. The supernatant was allowed to evaporate, and the residues were dissolved in methanol and stored at 4°C.

Thin layer chromatography analysis of 20 μL extracts spotted on a 20 × 10 cm glass silica^60^ F_254_ gel plate (Sigma, Canada) was performed as reported previously (Tambong and Höfte 2001). Synthetic phenazine‐1‐carboxylic acid and pyrrolnitrin (Sigma, Canada) were dissolved in methanol and used as positive controls. The mobile phase consisting of chloroform: acetic acid (49:1, v/v) in a glass chamber was used to separate the metabolites on the silica plate and run for 2 h. The silica plate was dried in a fume hood for 20 min at room temperature in the dark, followed by imaging on a Ultra‐Violet transilluminator (Spectroline, Fisher Scientific, Canada).

Bacterial extracts were analysed by UHPLC–MS on the LTQ‐orbitrap XL coupled to the Dionex Ultimate 3000 UHPLC. The chromatographic conditions were adopted from Brauer et al. (2024) with modifications as follows: Kinetex column (C_18_, 2.1 × 50 mm, 1.7 μm) maintained at 30°C with a 0.35 mL/min flow rate and LCMS grade water (Thermo Scientific, Fairlawn, NJ) as the mobile phase (A) and LCMS grade acetonitrile (Thermo Scientific, Fairlawn, NJ) as the mobile phase (B) each containing 0.1% formic acid, were used to resolve the compounds. The gradient program started with 5% B for 0.5 min, increased to 95% B over the 4.5 min, then held at 95% B for 8 min. Finally, the gradient returned to 5% B by 8.5 min and held for 3 min to equilibrate the column to starting conditions. High‐resolution mass spectral data were recorded in positive (ESI+) at a resolution of 30,000 and other parameters as previously reported by Brauer et al. (2024).

Whenever possible, metabolites were annotated by comparison of exact m/z (± 5 ppm), retention time and MS^2^ fragmentation pattern to authentic standards. As such, phenazine‐1‐carboxylic acid (PCA) with molecular formula C_13_H_8_N_2_O_2_ was annotated by comparing it with the authentic standard. PCA was detected as the [M + H]^+^ ions at *m/z 225*.0656 (m/z_cal_ 225.0663, Δm = 0.3 ppm) and as the sodium adduct [M + Na]^+^ ions at *m/z* 247.0473. Additionally, the fragment ions at *m/z* 207.0552 and *m/z* 179.0602 were observed as the loss of water [M‐H_2_O]^+^ and the loss of carboxylate [‐COOH] moiety, respectively, from the precursor ion of PCA. The Thermo XCalibur 2.2 software (ThermoFisher Scientific Inc., Waltham, MA) was used to accurately predict the molecular formulas of compounds with no available standards based on high‐resolution exact masses of [M + H]^+^ or [M + Na]^+^ ions combined with isotope abundance patterns, and the chromatogram of the extracted ion, as previously reported (Brauer et al. 2024). Under the specified conditions, PCA was eluted at 5.95 min. The peak at 5.25 min at *m/z* 197.0707 was annotated as a pseudo‐molecular ion [M + H]^+^ corresponding to the molecular formula C_12_H_8_ON_2_. This compound is 27.9948 amu less than PCA, and this mass difference corresponds to carbon monoxide CO. As a standard was not available and there was not enough pure compound to run the NMR to conclusively determine its structure, based on the exact mass, fragmentation pattern and database search, the compound is annotated as hydroxy phenazine. Similarly, a peak at 4.49 min at *m/z* = 245.1283 [M + H]^+^ corresponds to the molecular formula C_14_H_16_N_2_O_2_. Peaks at 4.10 and 3.55 min were the [M + H]^+^ ions at m/z = 211.1440 and *m/z* 197.1283 and correspond to the molecular formulas C_11_H_18_N_2_O_2_ and C_10_H_16_N_2_O_2_, respectively, suggesting the difference of a ‐CH_2_ group amongst the two compounds. Additionally, annotations of the unknown metabolites were also supported by the GC–MS run and associated National Institute of Standards and Technology (NIST) database match.

### 4.6 Phenazine and Pyrrolnitrin Gene Expression Studies

We analysed two key genes in each of the biosynthetic pathways with quantitative reverse‐transcriptase PCR (qRT‐PCR). The *phz*B and *phz*F genes were selected for the phenazine cluster and *prn*C and *prn*D for the pyrrolnitrin cluster. Total RNA was extracted from 1 mL of overnight bacterial cultures using the Nucleospin RNA extraction kit (Takara Bio, USA), according to the provided protocol. RNA quality was assessed using the Agilent 2100 bioanalyzer (Canada). RNA was reverse transcribed to complementary DNA (cDNA) using the Takara Bio RNA to cDNA EcoDry Premix (Oligo dT) as recommended by the manufacturer. qRT‐PCR amplifications were performed using the PerfeCTa SYBR green Fast Mix (Quantabio, Beverly, MA) and run in a BioRad Chromo4 Real‐Time PCR Detector. A total of 10 ng of cDNA was used as input for the qRT‐PCR reactions with forward‐reverse primer pairs (Table S1) designed using IDT PrimerQuest software and synthesised by IDT (Canada). The qPCR cycling parameters were an initial denaturation at 95°C for 30 s; 45 cycles of 45 s of denaturation at 95°C, 5 s; annealing at 60°C for 15 s; 72°C for 10 s, followed by a plate read. Melt curve analysis was performed within the 65°C to 95°C range, at 0.5°C increments. Real‐time PCR was performed in triplicate and relative gene expression was calculated using the formula 2−^ΔΔCt^ as previously reported (Livak et al. 2013). Ct values of selected genes were normalised to values of the *rpo*D housekeeping gene.

## Author Contributions

**Mercy Akuma:** investigation, validation, formal analysis, methodology, writing – original draft, visualization. **Sylvia Ighem Chi:** methodology, validation, investigation, formal analysis, writing – review and editing, visualization. **Renlin Xu:** formal analysis, investigation, methodology, writing – review and editing. **Indira Thapa:** visualization, formal analysis, methodology, writing – review and editing. **Barbara Blackwell:** formal analysis, visualization, methodology, writing – review and editing. **James Tabi Tambong:** conceptualization, funding acquisition, writing – original draft, writing – review and editing, formal analysis, project administration, resources, supervision, data curation, investigation, validation, visualization, methodology.

## Ethics Statement

The authors have nothing to report.

## Conflicts of Interest

The authors declare no conflicts of interest.

## Supporting information


**Figure S1.** *Pseudomonas chlororaphis* S1Bt23 Wild type (WT) and S1Bt23‐GFP streaked on Luria‐Bertani agar medium and observed under ultraviolet light.
**Figure S2.** Absence of the expected PCR amplicon of the green fluorescent gene in *Pseudomonas chlororaphis* S1Bt23 wild type (WT) (lane 1) but present in GFP‐transformant of S1Bt23 (lane 2). Expected size of GFP fragment is 633 bp. GeneRuler 100 bp Plus DNA Ladder (M) was used as a molecular weight marker. The primers used were specifically designed to target the *gfp* gene in the pPNptGreen plasmid.
**Figure S3.** Phenotype of *Pseudomonas chlororaphis* S1Bt23 Wild type (WT), S1Bt23‐pVSP61 and S1Bt23‐GFP streaked on Luria‐Bertani agar plate medium observed under white and ultraviolet lights. Note the fluorescence under UV due to the presence of the gfp gene in S1Bt23‐GFP.
**Figure S4.** Dual culture assays of *Pythium ultimum* and *Pseudomonas chlororaphis* strain S1Bt23‐pVSP61 (left) or green fluorescent protein‐transformed S1Bt23 (S1Bt23‐GFP) (right). Note the loss of antagonism on glucose‐casamino acid‐yeast agar medium inoculated with S1Bt23‐GFP, 5 days after incubation.
**Figure S5.** Thin‐layer chromatography (TLC) analysis of extracts from the supernatants (left) and cell pellets (right) of 1, phenazines or pyrrolnitrin standards; 2, wild type (WT) S1Bt23; 3, S1Bt23‐pVSP61; and 4, S1Bt23‐GFP for the presence of phenazine and pyrrolnitrin, respectively. Synthetic phenazine‐1‐carboxylic acid (PCA) and pyrrolnitrin standards were included. Phenazine visualisation was imaged under UV light and pyrrolnitrin under white light using Erhlich’s reagent spray. *Note the lack of band depicting either phenazine (dark blue band) or pyrrolnitrin (purple band) in S1Bt23‐GFP.
**Table S1.** Specific primers used in qPCR gene expression studies of phenazine and pyrrolnitrin biosynthetic clusters.

**Video S1.** Video shows active upward translocation of S1Bt23‐GFP cells in the xylem vessels. Hover the computer cursor over the video to see the play button.

## Data Availability

The data that support the findings of this study are available from the corresponding author upon reasonable request.
