# Supplementary material for: Plasmid pPNptGreen Expression of Green Fluorescent Protein in Pseudomonas chlororaphis Strain S1Bt23 Abrogates Biocontrol Activity Against Pythium ultimum
Source: Environ Microbiol Rep. 2025 Apr 16;17(2):e70083. doi: 10.1111/1758-2229.70083 (PMC12002801; doi:10.1111/1758-2229.70083)
Supplement: Supplementary file 1 — Figure S1. Pseudomonas chlororaphis S1Bt23 Wild type (WT) and S1Bt23‐GFP streaked on Luria‐Bertani agar medium and observed under ultraviolet light. Figure S2. Absence of the expected PCR amplicon of the green fluorescent gene in Pseudomonas chlororaphis S1Bt23 wild type (WT) (lane 1) but present in GFP‐transformant of S1Bt23 (lane 2). Expected size of GFP fragment is 633 bp. GeneRuler 100 bp Plus DNA Ladder (M) was used as a molecular weight marker. The primers used were specifically designed to target the gfp gene in the pPNptGreen plasmid. Figure S3. Phenotype of Pseudomonas chlororaphis S1Bt23 Wild type (WT), S1Bt23‐pVSP61 and S1Bt23‐GFP streaked on Luria‐Bertani agar plate medium observed under white and ultraviolet lights. Note the fluorescence under UV due to the presence of the gfp gene in S1Bt23‐GFP. Figure S4. Dual culture assays of Pythium ultimum and Pseudomonas chlororaphis strain S1Bt23‐pVSP61 (left) or green fluorescent protein‐transformed S1Bt23 (S1Bt23‐GFP) (right). Note the loss of antagonism on glucose‐casamino acid‐yeast agar medium inoculated with S1Bt23‐GFP, 5 days after incubation. Figure S5. Thin‐layer chromatography (TLC) analysis of extracts from the supernatants (left) and cell pellets (right) of 1, phenazines or pyrrolnitrin standards; 2, wild type (WT) S1Bt23; 3, S1Bt23‐pVSP61; and 4, S1Bt23‐GFP for the presence of phenazine and pyrrolnitrin, respectively. Synthetic phenazine‐1‐carboxylic acid (PCA) and pyrrolnitrin standards were included. Phenazine visualisation was imaged under UV light and pyrrolnitrin under white light using Erhlich’s reagent spray. *Note the lack of band depicting either phenazine (dark blue band) or pyrrolnitrin (purple band) in S1Bt23‐GFP. Table S1. Specific primers used in qPCR gene expression studies of phenazine and pyrrolnitrin biosynthetic clusters. [file EMI4-17-e70083-s002.pdf]

Plasmid pPNptGreen expression of Green Fluorescent Protein in *Pseudomonas chlororaphis* strain S1Bt23 abrogates biocontrol activity against *Pythium ultimum*

Mercy Akuma[1,2]†, Sylvia Ighem Chi[3]†, Renlin Xu[1], Indira Thapa[1], Barbara Blackwell[1] and James Tabi Tambong[1,4]*

[1] Ottawa Research and Development Centre, Agriculture and Agri-Food Canada, Ottawa, Ontario, K1A 0C6;
[2]University of Ottawa, Ottawa, Ontario, K1N 6N5;
[3]Canadian Blood Service, Ottawa, Ontario, K1G 4J5;
[4]Department of Plant Science, University of Manitoba, Winnipeg, MB. R3T 2N2

† equal contribution

*Corresponding author: James T. Tambong (james.tambong@agr.gc.ca)

Supplementary materials:

Table S1

Figure S1
Figure S2
Figure S3
Figure S4
Figure S5

Table S1. Specific primers used in qPCR gene expression studies of phenazine and pyrrolnitrin biosynthetic clusters

Gene	Biosythetic cluster	**Forward primer (5'-3')**	**Reverse primer (5'-3')**
*phz*B	Phenazines	GGGAGTGGTACAACGTCAAA	GCAGGAAGTGGTTCTCGTAATA
*phz*F	Phenazines	CGTCATCATCGACGCCTTT	GGTTTCCGACAGGTTCATCTC
*prn*C	Pyrrolnitrin	CTCTGGGTGATTCCGTTCAA	AAATGTCCGTTGTCGGGTAG
*prn*D	Pyrrolnitrin	CCTGAACATGTCGCAGATGAA	CACACACTGGAGCAACTTGTA
*rpo*D	Internal control	GGCAGATGTTGCAGGAAATG	CCATGGAGATCGGCTCTTTAG

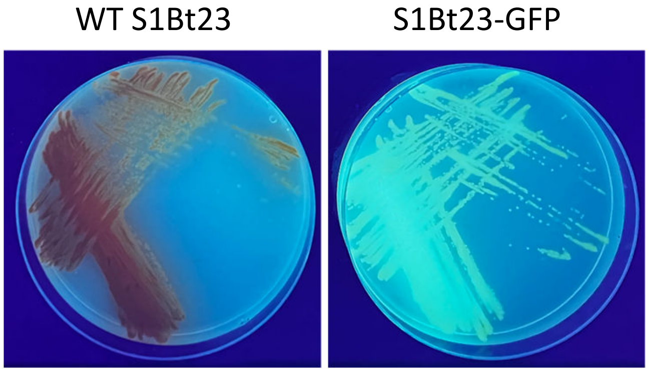

Figure S1. S1Bt23Wild type (WT) and S1Bt23-GFP streaked on Luria-Bertani agar medium and observed under ultraviolet light.

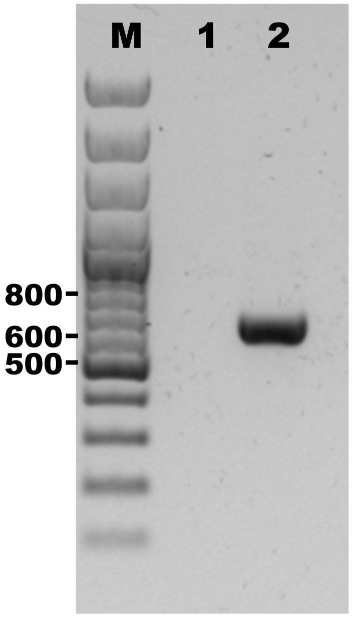

Figure S2. Absence of the expected PCR amplicon of the green fluorescent gene in *Pseudomonas chlororaphis* S1Bt23 wild type (WT) (**lane 1**) but present in GFP-transformant of S1Bt23 (**lane 2**) . Expected size of GFP fragment is 633 bp. GeneRuler 100 bp Plus DNA Ladder (**M**) was used as a molecular weight marker. The primers used were specifically designed to target the *gfp* gene in the pPNptGreen plasmid.

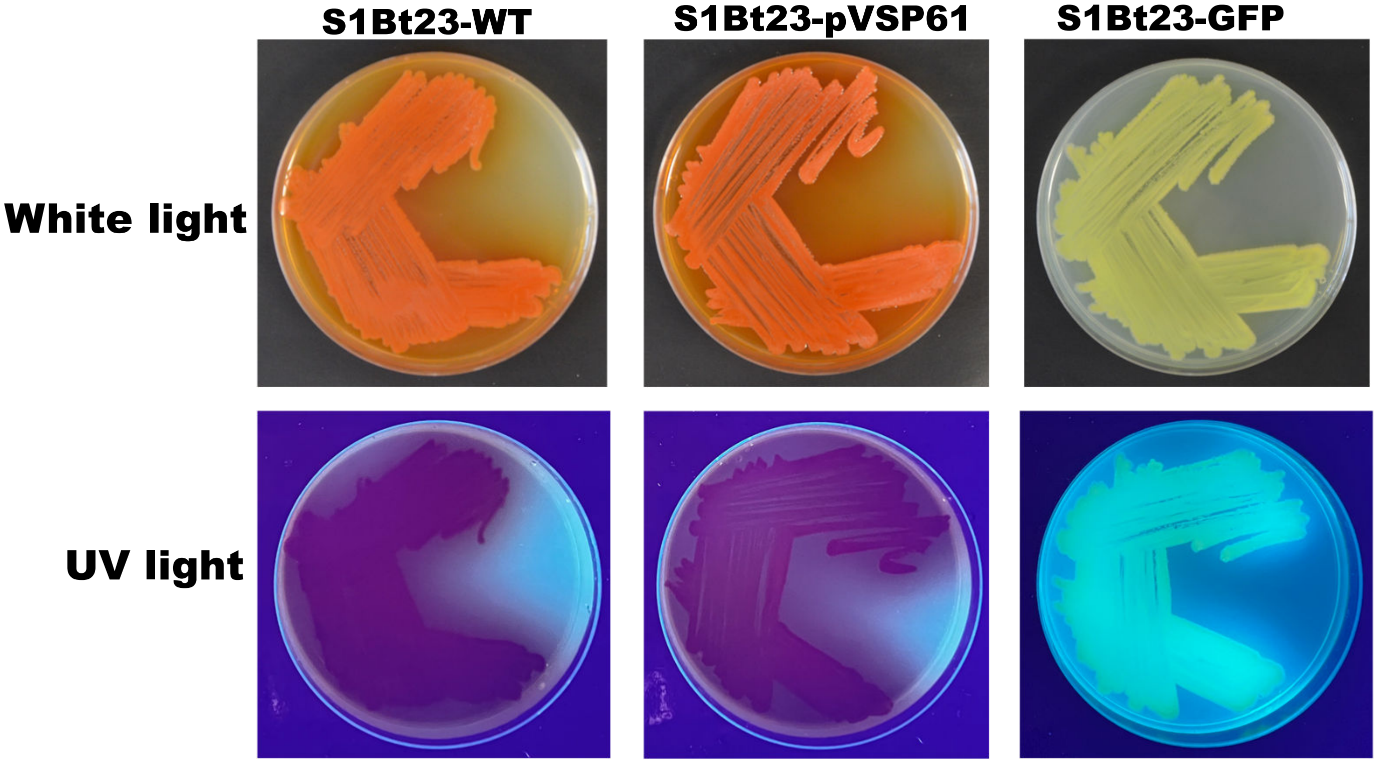

Figure S3. Phenotype of *Pseudomonas chlororaphis* S1Bt23 Wild type (WT), S1Bt23-pVSP61 and S1Bt23-GFP streaked on Luria-Bertani agar plate medium observed under white and ultraviolet lights. Note the fluorescence under UV due to the presence of the *gfp* gene in S1Bt23-GFP.

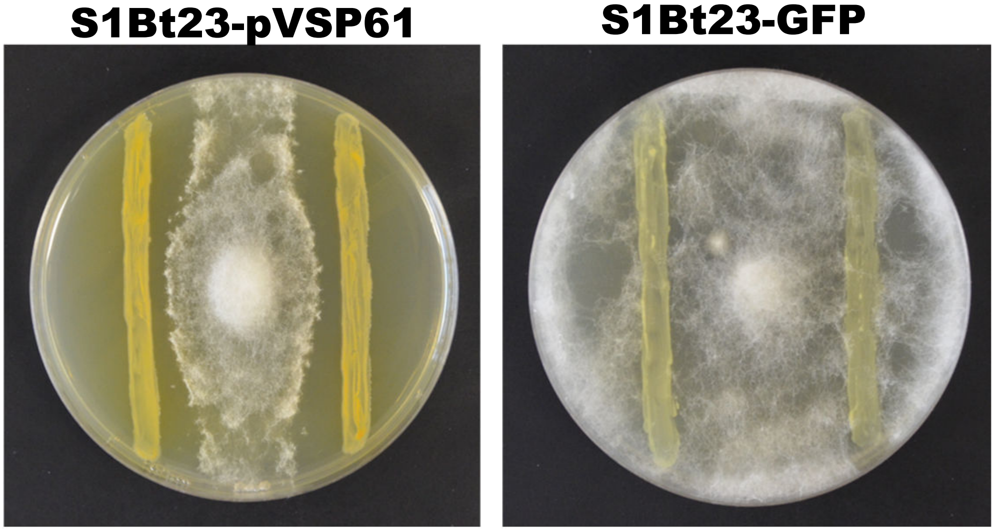

Figure S4. Dual culture assays of *Pythium ultimum* and *Pseudomonas chlororaphis* strain S1Bt23-pVSP61 (**left**) or green fluorescent protein-transformed S1Bt23 (S1Bt23-GFP)(**right**). Note the loss of antagonism on glucose-casamino acid-yeast agar medium inoculated with S1Bt23-GFP, 5 days after incubation.

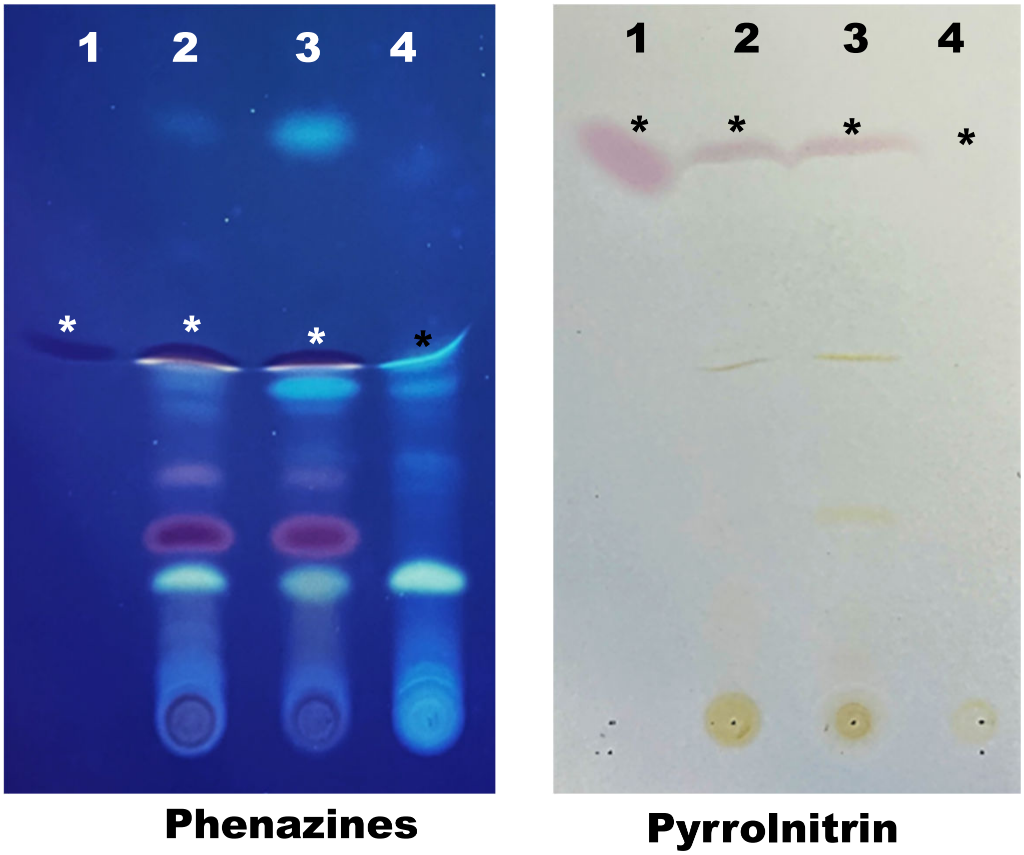

Figure S5. Thin-layer chromatography (TLC) analysis of extracts from the supernatants (**left**) and cell pellets (**right**) of 1, phenazines or pyrrolnitrin standards; 2, wild type (WT) S1Bt23; 3, S1Bt23-pVSP61; and 4, S1Bt23-GFP for the presence of phenazine and pyrrolnitrin, respectively. Synthetic phenazine-1-carboxylic acid (PCA) and pyrrolnitrin standards were included. Phenazine visualization was imaged under UV light and pyrrolnitrin under white light using Erhlich's reagent spray. *Note the lack of band depicting either phenazine (dark blue band) or pyrrolnitrin (purple band) in S1Bt23-GFP.
