# Supplementary material for: Plasmid pPNptGreen Expression of Green Fluorescent Protein in Pseudomonas chlororaphis Strain S1Bt23 Abrogates Biocontrol Activity Against Pythium ultimum
Source: Environ Microbiol Rep. 2025 Apr 16;17(2):e70083. doi: 10.1111/1758-2229.70083 (PMC12002801; doi:10.1111/1758-2229.70083)
Supplement: Supplementary file 2 — Video S1. Video shows active upward translocation of S1Bt23‐GFP cells in the xylem vessels. Hover the computer cursor over the video to see the play button. [file EMI4-17-e70083-s001.pptx]

## Slide 1
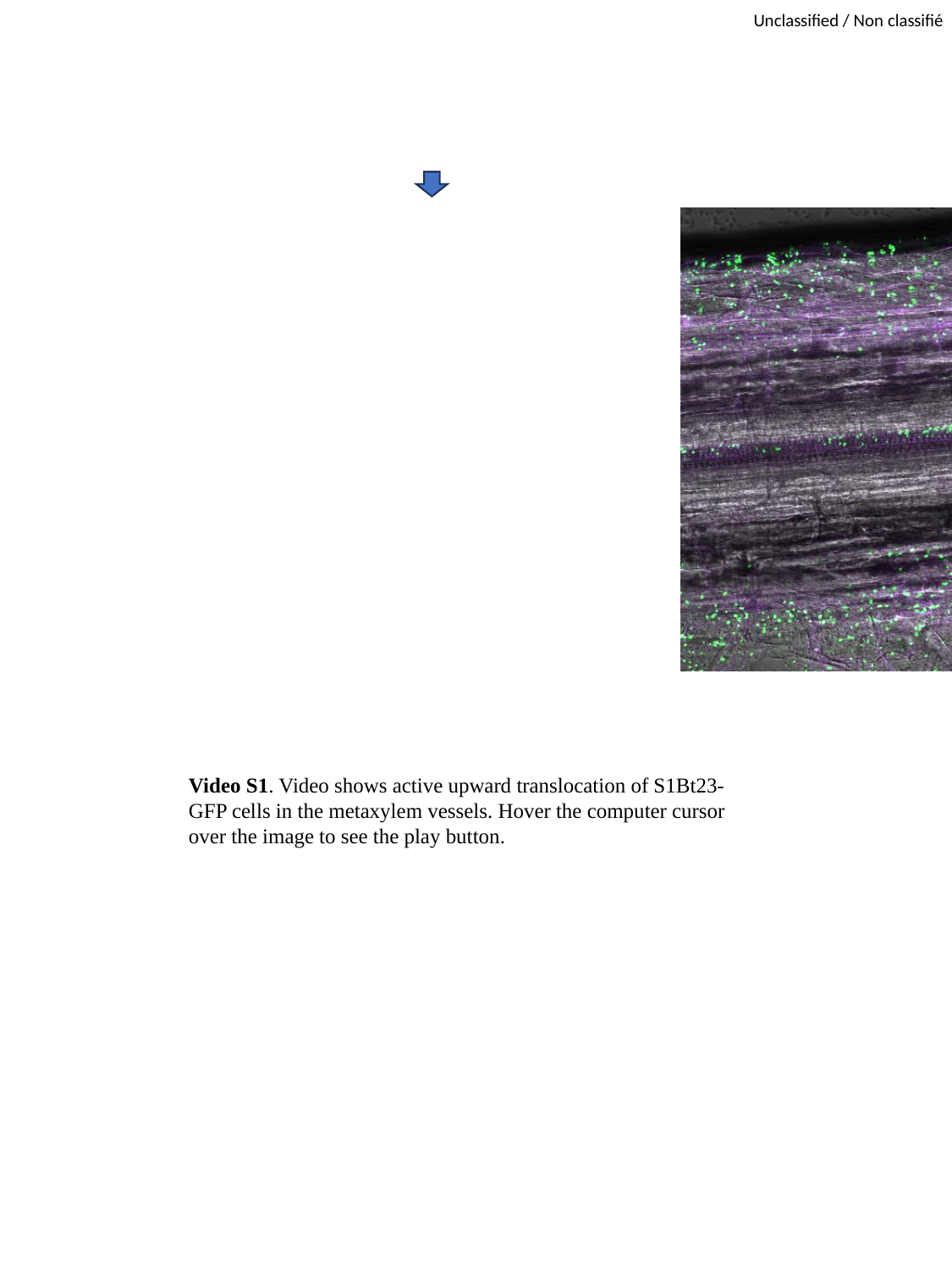

Video S1. Video shows active upward translocation of S1Bt23-GFP cells in the metaxylem vessels. Hover the computer cursor over the image to see the play button.
